# Development and implementation of the Caribbean Laboratory Quality Management Systems Stepwise Improvement Process (LQMS-SIP) Towards Accreditation

**DOI:** 10.4102/ajlm.v6i1.496

**Published:** 2017-02-24

**Authors:** George Alemnji, Lisa Edghill, Giselle Guevara, Sacha Wallace-Sankarsingh, Rachel Albalak, Sebastien Cognat, John Nkengasong, Jean-Marc Gabastou

**Affiliations:** 1 Centers for Disease Control and Prevention (CDC), Caribbean Regional Office, Bridgetown, Barbados; 2 Caribbean Public Health Agency (CARPHA), Port of Spain, Trinidad and Tobago; 3 World Health Organisation (WHO), Lyon, France; 4 US Centers for Disease Control and Prevention (CDC), Atlanta, Georgia, United States; 5 Pan American Health Organization (PAHO), Lima, Peru

## Abstract

**Background:**

Implementing quality management systems and accrediting laboratories in the Caribbean has been a challenge.

**Objectives:**

We report the development of a stepwise process for quality systems improvement in the Caribbean Region.

**Methods:**

The Caribbean Laboratory Stakeholders met under a joint Pan American Health Organization/US Centers for Disease Control and Prevention initiative and developed a user-friendly framework called ‘Laboratory Quality Management System – Stepwise Improvement Process (LQMS-SIP) Towards Accreditation’ to support countries in strengthening laboratory services through a stepwise approach toward fulfilling the ISO 15189: 2012 requirements.

**Results:**

This approach consists of a three-tiered framework. Tier 1 represents the minimum requirements corresponding to the mandatory criteria for obtaining a licence from the Ministry of Health of the participating country. The next two tiers are quality improvement milestones that are achieved through the implementation of specific quality management system requirements. Laboratories that meet the requirements of the three tiers will be encouraged to apply for accreditation. The Caribbean Regional Organisation for Standards and Quality hosts the LQMS-SIP Secretariat and will work with countries, including the Ministry of Health and stakeholders, including laboratory staff, to coordinate and implement LQMS-SIP activities. The Caribbean Public Health Agency will coordinate and advocate for the LQMS-SIP implementation.

**Conclusion:**

This article presents the Caribbean LQMS-SIP framework and describes how it will be implemented among various countries in the region to achieve quality improvement.

## Introduction

Strengthening laboratory services and systems to meet the challenge of the growing burden of diseases of global public health importance has been the focus of discussions and recommendations by the international community.^1,2,3^ Implementing laboratory quality management systems (QMS) and attaining accreditation has been an important part of these discussions.^4,5^ Laboratory accreditation to international standards verifies laboratories’ competence and ensures that they can provide evidence that test results are reliable and accurate to support patient care and public health practices.^6,7^

Strengthening health systems, especially medical laboratories, in the Caribbean has been a challenge. As a result, few public health laboratories are accredited. Several country and regional efforts have been made to improve quality systems and achieve accreditation of the laboratories in the region. For example, the Caribbean Epidemiology Centre/European Union-funded project, carried out between 2002–2007, designed and implemented a 30-month blended accredited training programme on laboratory quality systems improvement that graduated 40 senior laboratory staff, initiated the Caribbean Laboratory Accreditation Scheme, and recommended that the International Standard ISO 15189 be the recognised standard in the Caribbean for medical laboratories.^8^ Despite these efforts, reports indicate that by 2008 only nine (5.2%) of the estimated 173 laboratories in the Caribbean region were accredited. Six of these were medical laboratories.^9^ All of the accredited laboratories were either private or faith-based health facility laboratories. Thus, although more than 90% of the population utilises public or government laboratory services, none of these services were accredited.

Cognisant of these challenges, a joint collaboration between the Pan American Health Organization and the US Centers for Disease Control and Prevention, together with other regional stakeholders in 2010, decided to develop the Caribbean regional Laboratory Quality Management System – Stepwise Improvement Process (LQMS-SIP) Towards Accreditation framework to address laboratory quality challenges.^10^ This initiative aligns well with the World Health Organization (WHO) and the US Centers for Disease Control and Prevention Joint Conference on Laboratory Quality Systems held in April 2008 in Lyon, France, which recommended that ‘countries with limited resources consider taking a staged approach, where principal requirements for all are stated in the national laboratory standards as a minimum requirement, while more advanced and national reference laboratories are encouraged to aim at meeting internationally accepted standards such as ISO 15189’.^2^ Additionally, successful implementation of QMS and achievement of accreditation using the stepwise approach have been documented in other regions such as the WHO Regional Office for Africa’s (AFRO) Stepwise Laboratory Improvement Process Towards Accreditation (SLIPTA),^11,12^ the Thailand stepwise accreditation process,^13^ and the Argentina scheme.^14^ In this article, we present the Caribbean LQMS-SIP framework, developed following the above joint initiative. We also describe how the LQMS-SIP framework will be implemented among various stakeholders and countries to achieve a common goal.

## The Caribbean region

Rich in culture and diverse ethnicities, the Caribbean has the world’s largest collection of small island states, many of them independent nations. Many of them depend almost solely on tourism as the main economic driver, resulting in a variety of different growth rates and per capita incomes. A disease outbreak can have catastrophic impacts on the social and economic development of these already fragile economies, which can lead to global isolation. Evidence has shown that a priority for government is to ensure that the health sector, specifically medical laboratories, maintain a high capacity to provide reliable and timely information to protect public health and guarantee long-term sustainability of economic growth factors.

The Caribbean also has several cross-border regional organisations, unions, and groups that have been formed based on commonalities in culture, economy, religion, health specific needs, and the common good. These regional groupings have become very important as they leverage resources, reduce costs, and create a clear line of commitment and support to ensure that the smaller islands share and benefit from various opportunities and capacities found in the neighbouring larger islands. This is why a regional approach has been used in the development and implementation of the LQMS-SIP.

## LQMS-SIP: Implementation structure and stakeholders

The implementation of the LQMS-SIP is a joint effort by several stakeholders in the region, with each partner performing activities specific to its recognised role and providing expertise in the implementation process as required. Specifically, the Caribbean Community Regional Organisation for Standards and Quality, which is the Caribbean Community centre for promoting efficiency and competitive production in goods and services through standardising and verifying quality, has hosted the Secretariat of the LQMS-SIP since mid 2013. Stakeholders agreed, at a regional consultation in 2014, that the management of the LQMS-SIP will be a mutual cooperation and collaboration among the two recognised Caribbean National Accreditation Bodies and the National Accreditation Focal Points, based within the Bureau of Standards of each country, and the Caribbean Regional Organisation for Standards and Quality Secretariat. The Caribbean Public Health Agency, mandated to serve as a coordination unit for the Caribbean Public Health Laboratory Network, will continue this function to ensure coordination and collaboration of various stakeholders for a common interest. The Ministries of Health of various countries, recognised as the principal owners of the process, are expected to work closely with the above entities as they rollout and implement the LQMS-SIP in their various countries. Other stakeholders including the Pan American Health Organisation/WHO, the US Centers for Disease Control and Prevention, professional associations and non-government organisations will work to sustain functional Public Private Partnerships to advance the course and ensure the sustainability of the LQMS-SIP.^10^

## LQMS-SIP: Implementation process

### Eligibility, application, and enrolment

**FIGURE 1:**
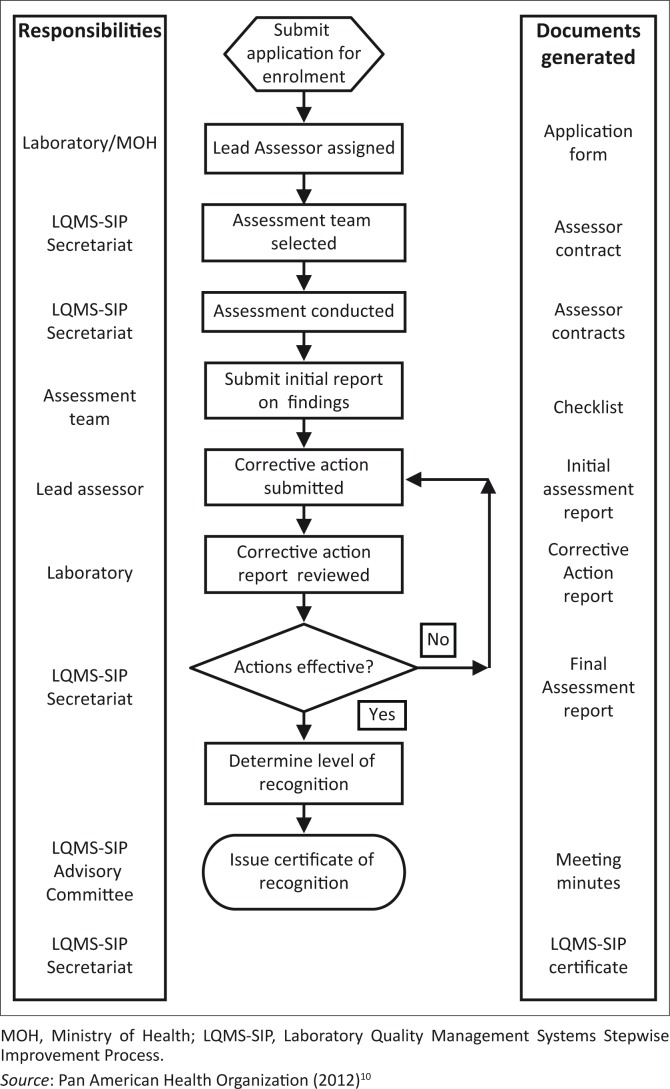
Flow chart of the Caribbean Laboratory Quality Management Systems Stepwise Improvement Process, showing the different steps for enrolment and participation of laboratories.

All laboratories (both public and private) are eligible to participate in the Caribbean LQMS-SIP. Application and enrolment will follow guidelines of national laboratory regulatory systems in participating countries and the LQMS-SIP Secretariat will receive and review all applications. A laboratory that applies to be evaluated for Tier 1 or higher will be sent an enrolment letter containing the enrolment date, enrolment number, and proposed timeframe for scheduling an assessment. Qualified assessors will then be selected based on the nature and type of laboratory. Monitoring of the process, including competence and performance of the assessors, logistics, and communication with the Ministry of Health and/or laboratory to agree on suitable dates for the assessment, will be coordinated by the LQMS-SIP Secretariat as outlined in Figure 1.^10^

### Checklist

The assessment will be carried out using the Caribbean LQMS-SIP checklist, which is on the ISO 15189:2012 standard and is broken down into the two main sections: management and technical requirements. The checklist is further subdivided into 15 management and 10 technical clauses; hence, the same structure as the ISO 15189 standard clause numbering system has been used to form the framework of the LQMS-SIP checklist categories (Table 1). An update was also made to add safety requirements based on the ISO 15190 standard, to ensure that each laboratory assessment gave a thorough review of the system. This checklist has a total of 104 requirements that have to be met by laboratories at the Tier 3 level.

**TABLE 1:** Checklist of the Caribbean Laboratory Quality Management Systems Stepwise Improvement Process, showing how laboratories will be scored following their quality improvements.

Section	Requirements	Number of Requirements at each Tier level		
		Tier 1	Tier 2	Tier 3
**Management Requirements**				
4.1	Organisation and Management Responsibility	8	9	9
4.2	Quality Management System	1	2	3
4.3	Document Control	2	2	2
4.4	Service Agreements	0	2	2
4.5	Examination by Referral Laboratories	1	2	2
4.6	External Services and Supplies	0	0	1
4.7	Advisory Services	0	0	1
4.8	Resolution of Complaints	0	1	1
4.9	Identification and Control of Nonconformities	0	0	1
4.10	Corrective Action	0	0	1
4.11	Preventive Action	0	0	1
4.12	Continual Improvement	0	0	1
4.13	Control of Records	1	1	1
4.14	Evaluation and Audits	2	6	8
4.15	Management Review	1	1	4
	Sub-total	16	26	38
**Technical Requirements**				
5.1	Personnel	4	9	9
5.2	Accommodation and Environmental Conditions	6	6	6
5.3	Laboratory Equipment, Reagents and Consumables	3	10	14
5.4	Pre-Examination Processes	7	11	11
5.5	Examination Processes	1	2	6
5.6	Ensuring Quality of Examination Results	2	8	9
5.7	Post-Examination Processes	2	2	2
5.8	Reporting of Results	1	3	3
5.9	Release of Results	0	2	3
5.10	Laboratory Information Management	0	2	3
	Sub-total	26	55	66
**Grand total**	**42**	**81**	**104**

*Source:* Original table taken from Caribbean Guidance, 2012 updated to ISO 15189:2012^10^

### The tiered structure

**FIGURE 2:**
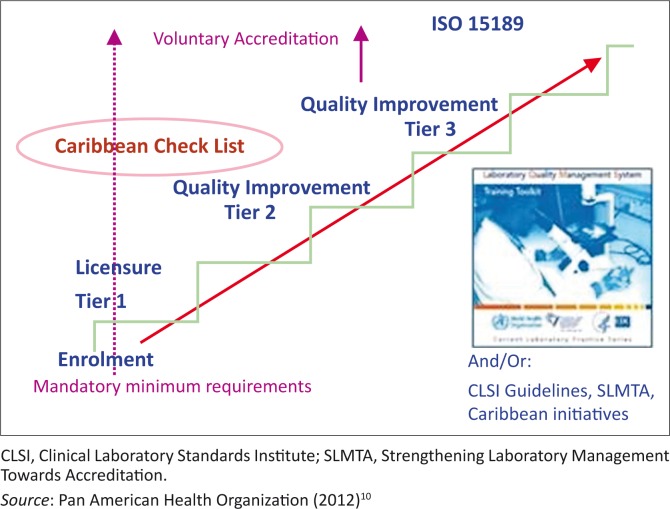
Caribbean Laboratory Quality Management Systems Stepwise Improvement Process showing the stepwise levels of recognition of laboratories at enrolment and following quality improvement.

Following each assessment, laboratories that meet the specific QMS requirements will be recognised according to the agreed three-tier (scoring) system (Figure 2). The basic national mandatory requirement for licensing of laboratories by the Ministry of Health is equivalent to Tier 1, while the quality improvement milestones that are acquired by fulfilling certain requirements of the ISO 15189 standard correspond to Tiers 2 and 3. Once a laboratory has successfully attained a tier level (1 or 2), they will be expected to continuously improve the QMS to meet the next level up at their follow up annual assessment. The National Accreditation focal points nominated by each Bureau of Standards will play an important role in linking the laboratory staff to human and technical resources to effectively eliminate the identified non-conformances and improve their performance. Laboratories attaining a Tier 3 certification at any time, will be encouraged to enrol in an established (internationally-recognised) accreditation programme based on ISO 15189: 2012 (Figure 2).^10^

### Mandatory Tier 1 requirements

The first tier will represent the minimum requirements corresponding to the mandatory criteria required for obtaining a national licence based on legislation enacted by the respective Ministries of Health (Figure 2). Ministries of Health are expected and encouraged to establish their own national regulatory systems for the operation of clinical laboratories and to build capacity for licensing in-country. At present, there are countries in the region which do not have operational systems for granting licences to laboratories. These Ministries of Health will be encouraged to enrol their laboratories in the LQMS-SIP while they work on national regulations for recognising Tier 1 as equivalent to a government license. Those countries which already inspect and grant licenses will be encouraged to review the requirements for such and subsequently align it to the criteria for Tier 1, since these are already based on the international standard. Where appropriate, the Ministries of Health should appoint a national accreditation focal point to coordinate all activities in-country, including engagement with the LQMS-SIP Secretariat.

### Laboratory assessment and recognition process

Once the assessment is completed, the assessors will submit their preliminary report to the laboratory and the final assessment report to the LQMS-SIP Secretariat. The laboratory will then be expected to respond to any gaps identified during the audit and to develop a customised corrective action plan within 30 days. Laboratories will be given up to 90 days to provide evidence of satisfactory resolution of non-conformities and improvement of the QMS. The assessors will review the non-conformances and produce a determination report. The advisory committee will be convened to review the determination report and recommend which tier level has been attained. The LQMS-SIP Secretariat will then make a final determination on awarding a certificate to the laboratory (Figure 1) for the relevant Tier achievement.

### Follow-up assessment and continuous quality improvement process

Laboratories that meet all the requirements of each tier will be issued a certificate of recognition that is valid for two years from the original date of issue. Application for reassessment and renewal of the certificate should be submitted to the LQMS-SIP Secretariat six months prior to the expiration of the existing certificate. Laboratories that obtain the Tier 3 recognition certificate are encouraged to apply for accreditation from a full member of the International Laboratory Accreditation Cooperation. Laboratories that are accredited to the ISO standard, will be transitioned from the LQMS-SIP assessment register and their achievement will be recognised on the Caribbean Regional Organisation for Standards and Quality website (http://www.crosq.org).

## Discussion

Well-designed and effective QMS operating in laboratories according to the requirements of an international standard such as ISO 15189, which could eventually lead to accreditation, is necessary for the Caribbean Region. The benefits of accreditation include the release of quality assured results for patients, cost savings in laboratory management, improved turn-around times, client satisfaction and enhanced staff competency.^6,4,7^ Furthermore, the International Health Regulations are an incentive for countries to develop, strengthen, and maintain the capacities of their laboratories to detect, assess, notify, and report health events to the WHO.^1^ Since countries in the Caribbean are signatory to this requirement, strengthening countries’ existing capacities for public health surveillance and quality laboratory services are critical for International Health Regulations implementation and continuous improvement of the health sector.

Quality systems improvement and accreditation of laboratories in the Caribbean has been very slow to develop, with very few clinical laboratories achieving this milestone. This includes the lack of advocacy, weak laboratory infrastructure and workforce, inadequate financial resources, and the complexity of the entire accreditation process.^8,9^ However, there are indications that using the stepwise approach, together with other evidence-based laboratory improvement tools in implementing QMS, could lead to more realistic results with minimal resources. For example, in 1994 the Argentine Biochemical Foundation created an accreditation programme for clinical laboratories that used a stepwise approach, starting from minimum requirements up to higher standards, that allowed laboratories to slowly begin the process of quality improvement and eventually reach the desired result.^14^ In 2002, the Latin American Confederation for Clinical Biochemistry (Confederación Latinoamericana de Bioquímica Clínica) and the Pan American Health Organisation/WHO developed a guideline for the development of medical laboratory accreditation tools in Latin America and a course on quality management and good laboratory practices,^15,16^ which includes checklists for a gradual implementation. Data from Lesotho in Africa show that using the WHO AFRO stepwise laboratory quality improvement programme,^17^ that also included the use of the Strengthening Laboratory Management Towards Accreditation training programme and mentorship, resulted in tangible improvements in the development and implementation of quality systems in the enrolled laboratories.^11^ Therefore, developing and using the stepwise-based Caribbean LQMS-SIP for countries in the region has the potential to improve quality systems implementation and to result in eventual accreditation of more laboratories.

An important component of the stepwise process for laboratory QMS implementation towards accreditation is that it provides an opportunity for measuring laboratory progress and identifying and improving quality systems in real-time. It also provides an opportunity for recognising gains in competence through periodic assessments and award of recognition certificates to laboratories as they make progress toward fulfilling the requirements of the standard. This interactive approach makes the entire process more dynamic and encouraging and has the potential to inspire more laboratories in the region to initiate and continue the process of quality improvement toward accreditation.

Although the stepwise approach for quality systems improvement has proven to be very helpful, it is necessary to tailor it to the laboratory infrastructure and capacity of each region or specific country, as tools developed in different settings may not address the immediate laboratory needs of other environments. Thailand developed a customised laboratory standard derived from multiple requirements contained in various international standards, but retained the most relevant and critical portions and made the standard applicable to Thailand and its infrastructure. The requirements of this standard were affordable, measureable, adaptable, sustainable and effective, and resulted in many laboratories in the country being accredited.^13^ The WHO AFRO SLIPTA programme,^17^ tailored to the needs of laboratories in Africa, resulted in many laboratories being enrolled in the quality systems improvement and accreditation process.^18^ The Caribbean LQMS-SIP was developed based on the needs and specific challenges of laboratories in the Caribbean. There are a number of unique or innovative features of the Caribbean scheme compared to systems that have been developed in other regions. The Caribbean scheme, for example, has three tiers compared to the five-star rating of the WHO AFRO process. Furthermore, the Caribbean scheme has licensing/regulation as a baseline requirement of its Tier 1, which is not common with the other systems. Hence, this scheme is specifically designed to address laboratory regulatory and quality issues within the Caribbean context.

Monitoring laboratory operations and ensuring compliance with national and international standards in many developing countries has been hampered by the lack of policies and regulations that empower governments to directly supervise laboratory operations.^19,20^ In some instances, this has resulted in using unqualified persons for laboratory operations, substandard testing, and release of unacceptable results meant for patient care and policy decision making.^21,22^ Past review of legislation developed and implemented among select countries in the Caribbean showed that there is still considerable work to be done toward establishing and instituting legislation for quality assurance in public health and clinical laboratories in many of these countries.^23^ As a result of this finding, it has been proposed that Tier 1 of the LQMS-SIP be mandatory and correspond to the requirement for being granted a licence based on legislation enacted by participating countries. Ministries of Health are encouraged to establish their own regulatory systems for operation of medical laboratories and build capacity for licensing by the government. Only laboratories – public or private – registered with the Ministry of Health and/or licenced in-country, will be allowed to enrol in the LQMS-SIP. Hence, fulfilment of the requirement of Tier 1 is considered an entry point for a laboratory to participate in the LQMS-SIP. This will minimise the number of incorrect medical laboratory practices in the Caribbean. To ensure that this framework is supported at all levels, quality improvement and the accreditation process should be well articulated in national health laboratory policies and strategic plans. More advocacy and sensitisation campaigns among policy makers, ministry officials, laboratory management and staff on the benefits of implementing an effective and sustainable QMS and attaining accreditation are needed.

### Limitations of implementing LQMS-SIP in the Caribbean

The implementation of the stepwise process for quality systems improvement and accreditation is new in the Caribbean. Some challenges include the lack of understanding of the process, limited support from laboratory staff and policy makers, and inadequate human, financial, and infrastructure resources. It is recommended that laboratory stakeholders develop more advocacy and awareness strategies that target policy makers and laboratory staff as well as strategies to raise funds to support infrastructure upgrades and human capacity development to meet the basic requirements of LQMS-SIP.

### Conclusion

The Caribbean LQMS-SIP framework is a three-tiered process developed to provide a user-friendly approach to implement QMS towards laboratory accreditation. Recognition certificates will be awarded as laboratories move from one tier to the next. Laboratories that reach Tier 3 will be encouraged to apply for accreditation from a recognised body. Governments are urged to take greater responsibility and to ensure that structures are in place in-country for licensing of medical laboratories and for their enrolment and participation in the LQMS-SIP. There is evidence that the stepwise accreditation process provides an opportunity for measuring laboratory progress and for recognising gains in competence as well as providing opportunities for identifying and improving quality systems. Hence, the LQMS-SIP has the potential to accelerate the process of quality systems improvement and laboratory accreditation in the region.

BOX 1 Lessons Learned.

- In resource-limited settings, using a simple stepwise approach to laboratory quality management can result in tangible improvements.
- The ISO 15189 international standard provides the minimum requirements for quality improvement, which can be applied in clinical laboratories, regardless of size and location.
- Scheduled laboratory assessments using a detailed checklist provide a simple but effective way to monitor progress when developing a quality management system.
